# “The One Time You Have Control over What They Eat”: A Qualitative Exploration of Mothers’ Practices to Establish Healthy Eating Behaviours during Weaning

**DOI:** 10.3390/nu11030562

**Published:** 2019-03-06

**Authors:** Eleni Spyreli, Michelle C. McKinley, Virginia Allen-Walker, Louise Tully, Jayne V. Woodside, Colette Kelly, Moira Dean

**Affiliations:** 1 Institute for Global Food Security, School of Biological Sciences, Queen’s University Belfast, Belfast BT9 5DL, UK; espyreli01@qub.ac.uk; 2 Nutrition Research Group, Centre for Public Health, Queen’s University Belfast, Belfast BT12 6BJ, UK; m.mckinley@qub.ac.uk (M.C.M.); vallenwalker01@qub.ac.uk (V.A.-W.); j.woodside@qub.ac.uk (J.V.W.); 3 Division of Population Health Sciences, Royal College of Surgeons, Dublin, Ireland; louisetully@rcsi.ie; 4 Health Promotion Research Centre, School of Health Sciences, NUI Galway, Ireland; colette.kelly@nuigalway.ie

**Keywords:** weaning, feeding practices, eating behaviours, diet diversity

## Abstract

Background: Weaning marks the transition from a milk-only diet to the consumption of solid foods. It is a time period where nutrition holds an undeniable importance and taste experiences have a long-lasting effect on food preferences. The factors and conditions that form parental feeding practices are yet to be fully understood; doing so can help target problematic behaviours and develop interventions aiming to modify them. Objective: This study used a qualitative methodology to gain a better understanding of parental experiences of weaning a child. Particular emphasis was placed on exploring the factors and conditions that favour the establishment of a healthy relationship with food in infancy and those that impede it. Methods: Thirty-seven mothers of healthy infants 3–14 months with no previous history of allergies or food-related disorders were recruited. Eight semi-structured focus group discussions were conducted, transcribed and analysed thematically. Results: Discussions revealed a number of opportunities to establish healthy eating habits during weaning, as well as relevant challenges. Important opportunities included: acting as a role model for healthy foods; giving multiple opportunities to try a food; food variety “so you don’t have a fussy eater”; and without food variety “things aren’t going to work properly”. Additionally, some of the challenges identified were: misconceptions about the definition of food variety; and distractions occurring during feeding. Conclusions: Mothers were mindful of the need to provide their children with appropriate nutritional stimuli during weaning. They were aware of their role in influencing their infants’ likes and used strategies such as modelling and repeated food exposure. The importance of a diverse diet in infancy was acknowledged, although knowledge gaps exist in relation to its definition. Distractions were tactfully employed by mothers to assist feeding. Findings of this study have applications in developing interventions for nutritional education in the complementary feeding period.

## 1. Introduction

Weaning is an important milestone in an infant’s development. It is defined as the transition from a milk-only diet to the consumption of family foods and it is also known as complementary feeding [1]. Nutrition has a dominant role in a child’s development during this time period for a number of reasons. Primarily, weaning is a period of rapid growth; as a result, the infant’s nutrient needs increase and stores acquired through in-utero life gradually deplete [2]. Additionally, diet and growth during weaning seem to have a programming effect for health outcomes later in life, as previous research demonstrates. With particular respect to adiposity, studies highlight that infants that have a rapid weight gain are more likely to become overweight or obese children and adults [3,4,5]. Moreover, the post-natal period is critical for brain development with environmental factors, including food, influencing both structural and functional aspects of the brain [6]. Taste experiences early in life can also affect later food preferences and thus food related behaviours [7,8].

Even though the first foods are eaten during weaning, the first flavour experiences occur earlier in life. Humans come to contact with smell and taste initially in the womb [9]. The first exposure to food is through the mother’s diet during pregnancy and, hence, children’ first preferences can be shaped by her food choices. Further exposure to foods after birth is through breast milk; an infant is more likely to show preference for foods which their mothers consumed often when breastfeeding. [10,11]. Moreover, humans innately prefer foods that taste sweet and hold an aversion towards those that taste bitter [12,13,14]. Similarly, for infants with no prior exposure to food (or milk), naturally sweet foods would elicit a better hedonic response in comparison to bitter or sour foods. Taking this inherent aversion into consideration, along with the fact that several nutritious foods taste bitter (e.g., green vegetables), health professionals advise that repeated exposure to these foods and serving them alongside sweet foods can encourage consumption in infancy [15,16]. 

Even in early infancy, children communicate enjoyment and refusal after exposure to a food. Responses can be expressed through head orientation and facial expressions (hedonic or non-hedonic). As far as facial expressions are concerned, a number of facial expressions have been associated with distinct tastes. Steiner and colleagues first described features displayed in faces of neonates after various sensory stimuli: lip thinning and lip licking with sweet taste; eyes closing and nose wrinkling with sour; and upper-lip elevating and gaping with bitter [17]. A number of well-designed experimental studies assessed mothers’ judgement of their infants’ liking to certain foods based on their facial responses [15,18,19]. Qualitative data would also be useful in providing an insight in parents’ thought process and practices in response to infants’ facial expressions, but currently such data is lacking. 

The Department of Health, in accordance with the World Health Organisation, advises that from six months a variety of complementary foods should be introduced alongside continued breastfeeding and/or breast milk substitutes [20,21]. Current parental practices in the UK however deviate from the recommendation in terms of providing a wide selection of appropriate complementary foods, as shown by the latest Diet and Nutrition Survey for Infants and Young Children (DNSIYC) [22]. 34% of the parents with a child aged 7–9 months avoided giving all meat/poultry/fish/seafood and the main reason was that these foods were not cooked in the household. Additionally, 9% of parents with a child in the same age group avoided giving fresh fruit and 7% salad vegetables (lettuce-tomato-cucumber).

Weaning can be an emotive and challenging time for parents, as they have a special role in helping their children shape their dietary behaviour through their feeding practices. The factors and conditions that form parental feeding practices are yet to be fully understood; doing so can help target any problematic weaning practices and modify them. To address this gap, this study set out to use qualitative methodology to gain a better understanding of parental experiences of weaning a child and more specifically to explore the factors and conditions that favour establishing a healthy relationship to food in infancy and those that impede it. It is hoped that ultimately, findings of this study will inform future interventions that provide nutritional education in the complementary feeding period.

## 2. Materials and Methods 

### 2.1. Study Design and Ethics

A qualitative study, using a semi-structured topic guide, was conducted with focus groups as the mode of data collection. This study formed part of a larger study across the island of Ireland exploring parental experiences of weaning and use of guidelines [23]. The focus of the present study was to identify mothers’ perceptions on acquisition of taste preferences and on the importance of food variety and their attitudes towards food exposure and shaping the feeding environment in weaning. This paper focuses exclusively on the introduction of solid foods as opposed to milk feeding and the terms ‘weaning’ and ‘complementary feeding’ will be used interchangeably.

Focus groups were selected as the data collection approach over alternative ones (e.g., individual interviews) due to their dynamic nature; they foster a group interaction which in turn can provide better insights into participants’ beliefs and experiences. Group members find themselves influenced by their co-speakers’ comments, respond with their ideas and make comparisons. In this way focus groups automatically highlight issues of consensus and diversity, something that doesn’t occur in individual interviews. Additionally, conducting focus groups saves time; groups produce data from a certain number of participants faster than individual interviews [24].

Ethical approval for the study was obtained by the School of Medicine, Dentistry and Biomedical Sciences Research Ethics Committee, Queen’s University Belfast. After being briefed on the study, participants consented to take part by signing a form that confirmed voluntary participation, confidentiality and data protection. 

### 2.2. Participant Selection 

Eligible participants were adult parents and legal guardians of healthy infants aged between 3 and 14 months who were able to read and converse in English. Participants from all genders were eligible. Even though weaning is not recommended till the 6th month, parents with children in a younger age were also included, as they might be already thinking of introducing solids and thus, can talk about their intentions. Prematurely born children, children with diagnosed allergies and intolerances and any medical condition that affects the ability to feed, swallow or digest were excluded from participating. Eligibility in the study was assessed with a screening questionnaire that was administered during recruitment. The sample size was to be determined by the interviewers when data saturation would be reached. 

Recruitment in the study utilised a purposive, snowball sampling technique and targeted community organisations in Northern Ireland that already engaged with the target population. The research team identified parent–toddler groups, community centres that offer programmes to young women and Sure Start centres, and established contact with the people who run them (gatekeepers). Sure Start centres are UK Government initiatives that provide help and advice on child and family health and parenting. Recruitment took place in the greater Belfast area and two areas outside Belfast (Armagh and Antrim), Northern Ireland, in order to capture the views of participants from urban as well as rural environments. 

Contact with gatekeepers was established and a quick overview of the study given. When the gatekeepers deemed recruitment in their organisation feasible and worthwhile, a visit from the research team was organised. The purpose of the visit was to describe the study and invite mothers to take part. Mothers were also encouraged to disseminate the call for participation to peers who might be interested. The screening questionnaires were completed by any mothers who expressed an interest in participating. Questionnaires were collected from the mothers and were reviewed for eligibility by the research team when they returned to the University. Participant inclusion/exclusion was determined and communicated to all those that showed an interest in participating. 

To maximise participation in the study, all participants were given a ‘One-4-all’ gift card worth £20 as a token of appreciation for their time and contribution, as well as educational material on weaning [25]. They were also given the option to be reimbursed for their travel expenses to and from the focus groups. 

### 2.3. Data Collection

Data collection took place in the organisations where participants were recruited from, as they were familiar and convenient for the participants. Focus groups were also organised when the usual parent groups would take place so that participants didn’t need to make special arrangements to attend. A reminder text was sent the day before the focus group was due to take place. 

Data were collected through focus groups which had the form of semi-structured conversations with open-ended, discussion-stimulating questions. The questions had been carefully considered by the research team in order to efficiently address the specific objectives of this project. Moreover, a review of the qualitative literature on weaning was performed to ensure the choice of under-explored themes in the topic guide. 

Images were also used to stimulate conversation where it didn’t occur naturally and to further elicit participants’ views. The images had been published in a study assessing fruit and vegetable acceptance in infancy [15] and permission was obtained to use during the focus groups. The images presented illustrated common components of responses to sour and bitter taste. 

The topic guide was piloted with the first focus group (attendees in it were included in the overall study sample) to assess clarity of questions and flow from one topic to the next. Minor adaptations on wording were made to the discussion guide as a result of the pilot (Table 1). The person who facilitated the discussions encouraged everyone’s contribution and probed when it was felt that the participants did not elaborate enough on a topic. When new ideas arose that were not part of the guide, the facilitator deviated from the script and probed accordingly. 

Two researchers, trained in focus group facilitation and data collection, were present at all focus groups: a doctoral researcher and dietitian, and a postdoctoral researcher and health psychologist. One facilitated the discussions and one took notes of participants’ discussions and their non-verbal responses, alternating between each other for every group. All conversations were audio recorded with two digital recorders. Before commencing with the topic guide, the facilitator gave a brief introduction reminding the participants of the audio recorders and the need to talk clearly and loud enough, the informal nature of the group discussions and study confidentiality. Demographic characteristics and infant feeding information were also recorded. Mothers with more than one children were asked to talk about their youngest child. 

Data collection ran in tandem with recruitment which started at the beginning of March 2017 and continued until the beginning of July 2017. The recordings were transcribed verbatim by a professional transcribing company and then proof-read by both facilitators to ensure accuracy. Participants were anonymised and given a unique study ID which consisted of the number of the focus group and their participant number represented as “Participant (focus group number)_(participant number)”. 

### 2.4. Data Analysis

All transcripts were imported and processed in NVivo 12 Pro software (QSR International Pty Ltd, Doncaster, Victoria, Australia). All data were coded by the first author who was previously trained on the steps of qualitative data analysis. Having Braun and Clarke [26] as a guide, thematic analysis was used to identify themes within the data. Accordingly, transcripts were read thoroughly so that familiarity with the data could be achieved. Initial codes were generated in a data-driven way; the content of the entire data set was coded without using a pre-existing coding frame. Two transcripts were coded independently by M.D. (psychologist) and the codes generated by the two researchers compared. Comparison between coding content demonstrated 90% similarity (inter-rater reliability); differences were discussed and resolved. The codes were grouped into key themes which were subsequently reviewed and refined to make sure that the themes were coherent and distinct from each other. Representative quotes were extracted from the data to illustrate typical views within each theme. At this stage the transcripts were re-read to ensure that the final themes accurately reflect the data. The process of data analysis and theming was supervised by and discussed with M.D. & M.M.K. Participant demographic characteristics and infant feeding information were analysed using IBM SPSS Statistics for Windows, Version 22.0 (IBM Corp. Armonk, NY, USA).

## 3. Results

### 3.1. Participation and Sample Characteristics

Eight focus group discussions took place with an average duration of 45 min. Six of them were conducted in Belfast and two in rural areas, Antrim and Armagh. Following the completion of the 6th focus group, the interviewers felt that data saturation was achieved and no new information was emerging and thus data collection was continued in two final focus groups.

Even though the call for participation was open to both male and female parents, only women came forward. Their demographic characteristics are presented in Table 2. Forty-five women were screened; they were given information on the study and the researchers took their details for further contact. Eight of them didn’t continue with the study. Reasons for not participating were: family member (including infant) being unwell; not eligible due to exclusion criteria; not interested in taking part anymore; and unable to reach on phone.

Thirty-seven women (mean age: 30.3 ± 6 years) took part in the discussions with an average of 5 participants per group (range: 3–6). Sixteen women were first-time mothers with the remainder reporting having between 1 and 3 or more other children. A summary of participants’ demographic characteristics is presented in Table 2. 

Slightly more than half of the participants (57%) breastfed at some point post-partum. Thirty mothers had already weaned their children at the time of the focus group (81%) and the remaining 7 were still offering a milk-only diet. Of the mothers who had weaned their infants, 47% (*n* = 14) had done so between 4 and 6 months, whereas a third (30%, *n* = 9) had started weaning before the 4th month and 23% of the overall sample had weaned according to the WHO guidelines at 6 months (*n* = 7). Age of introduction to solid foods was similar between breastfed and formula-fed children. Thirty-two per cent of mothers offered cereal products including baby rice as first foods, with a combination of fruit and vegetables being the next most popular option (22%).

### 3.2. Emerging Themes

Here this paper presents the qualitative findings, as they emerged from the focus groups. The conversations with mothers revealed a series of experiences that enabled them to encourage the development of positive eating habits by their children (Table 3). Several experiences worked in the opposite way and therefore, they were classed as challenges to establish healthy eating habits in infancy.

### 3.3. Opportunities to Shape a Healthy Relationship with Food

The importance of establishing balanced eating behaviours during the complementary feeding period was acknowledged by all mothers. Mothers perceived the first year of life to be a crucial time for children’s exposure to different foods and flavours and the idea of ‘getting as many different foods as possible’ was discussed. Ultimately, weaning was regarded as “the one time in their life you have complete control over what they eat” (Participant 007_03). The relevant themes that emerged are described below. 

#### 3.3.1. Acting as a Role Model for Healthy Foods

Mothers viewed their contribution in the feeding process as a primary force shaping children’s food preferences and habits. One way of achieving this was through modelling, as mothers had noticed that their infants exhibited a liking for the foods that they consumed. The affinity of the infants for their mum’s foods was often used as a way of dealing with infant’s food refusals and to introduce foods that mums wanted their children to eat.

- Participant 004_01: … now I’ve started trying to eat what, what I want him to eat so like I’ll have the banana too and he’ll eat it a lot better …

The awareness of their influence on the infants’ behaviour, along with the need to provide healthy meals, urged mothers to improve their own dietary choices. This was a positive strategy where the mum and infant influenced each other’s food choices.

- Participant 004_03: If she wasn’t here, we would be like throwing something into the microwave where it’s really conscious to cook, so I cook what’s suitable for her and then we have that, so it helps us eat healthily.

#### 3.3.2. Using Covert Approaches to Feed

Infants’ limited awareness of what’s on the plate was also a favourable condition for mothers to feed various healthy foods; particularly foods that the child had initially rejected. Women talked about hiding the undesired food in a liked one or modifying the consistency of that food.

- Participant 002_03: Bring something that they did like, then just switch the spoon back …

Moreover, mothers realised that their non-verbal communication during feeding is important and that it influences their infant’s attitudes towards a certain food. Creating an encouraging feeding atmosphere was helpful, whereas mum’s discouraging facial expressions lessened the child’s interest in the food.

- Participant 004_02: … so I would just try to kind of even get her bigger sister to be like ‘aww you’re doing really well’ … And all of us are sitting “oh well done” all being very encouraging.
- Participant 007_05: Your face if you’re giving it to them, if you don’t like it, they can see you like your face I think you wouldn’t be as like keen.

#### 3.3.3. Giving Multiple Opportunities to Try a Food

Gaining familiarity with a food through repeated exposure to it was utilised to a great extend too; particularly with the foods that were not loved by the child straight away. The frequency that a food was given was seen as a determining factor for a child to get used to this food and eventually enjoy it.

- Participant 008_01: … if you kept trying them until they actually, you know, acquired that taste for it cause the reason I tried the first time not like it you just put it to the side but if you keep trying like that taste will come for it.

#### 3.3.4. “It Starts in the Womb”

It was acknowledged that the opportunity for a mother to instil in her child good eating habits arises earlier than the time of weaning. A few participants talked about a mum’s contribution to the child’s sensory stimuli during pregnancy and lactation. Even though the idea was voiced without relevant probing, there were doubts about its validity.

- Participant 001_01: They say it starts in the womb, don’t they? … Especially, I was told especially strong flavours like garlic and curries and stuff like that it starts in the womb and then if you breast feed it’s meant to happen then as well.
- Participant 004_01: Sometimes I read too that what you ate in pregnancy can affect what your baby eats but I don’t know if that’s the thing or not …

#### 3.3.5. Facial Expressions not being Indicative of Food Rejections 

Participants were given a task of interpreting facial expressions of infants’ photographs. The photographs showed images of children’s facial responses to non-sweet tastes. The task was presented in order to elicit participants’ interpretations of non-hedonic responses to food. It was also an exercise to see how infants’ expressions of distaste may affect mothers’ feeding practices. Eventually, this exercise offered an opportunity to reveal that mothers were not discouraged by their children’s facial cues from offering the foods that triggered these cues. When presented with the images, the various facial expressions were mostly interpreted as occurring outside the food context placing the child in various moods.

- Participant 002_05: B … I wouldn’t interpret that as he doesn’t want the food.
- Participant 002_04: he looks quite content.
- Participant 002_02: The last one maybe she wants sleep.

In relation to feeding practices mothers explained that these facial responses did not necessarily indicate rejection of the food offered and mothers were aware that expressions of perceived distaste were common and did not necessarily mean that the infant didn’t wish to continue eating their meal. In similar situations at home, this attitude enabled them to carry on with the feeding until the desired food was accepted by their child.

- Participant 005_04: … Like he will screw his face up the first like three (spoonfuls) and then from that he would be fine and he does it with every single thing he eats. It’s not just, you think he doesn’t like it, but he does.
- Participant 008_03: Sometimes even when [infant’s name] likes her food she screws her face up just the first thing she’ll be like urgh and then she’s like mmm!

#### 3.3.6. Food Variety “So You Don’t Have a Fussy Eater”

Participants talked about the benefits of food variety as motivators to encourage a liking to a diverse eating pattern by their children. Supporting arguments were formulated with varied eloquence relating to the enjoyment of food and prevention of fussiness. A diverse diet in infancy was viewed to help prevent picky eating behaviours, whereas providing a limited food selection was associated with the consumption of monotonous diets and the reluctance to try unfamiliar foods in older ages.

- Participant 002_04: … you’re going to be really fussy if you don’t introduce it (variety) so they’ll just end up eating chips their whole life …

Concerns of having a fussy child in the family was a dominant topic in the focus groups. Having a fussy child was linked to increased stress during mealtimes. Also participants drew on their own experiences and concluded that being picky with food will be an inconvenience for the children themselves when they grow up, as it will prevent them from having an enjoyable eating experience.

- Participant 003_04: … I don’t think there’s anything worse than a fussy child, when it’s you know I don’t like that I don’t want it I don’t want it, it can get you really stressed …
- Participant 005_03: … I’m not a big fish eater, I’m more chicken … So it’s like some places I would go where there’s a set menu and sometimes there’s no chicken, so I’m just stuck eating soup and I think oh I kind of want to get [infant’s name] into like kind of eating everything …

#### 3.3.7. Without Food Variety “Things Aren’t Going to Work Properly”

In addition to the previous points on food variety, feeding the weaning infants with a wide range of foods was perceived to contribute to a sufficient intake of essential nutrients. Consequently, food exposure was believed to be responsible for a number of health issues. Participants were aware of the link between poor dietary patterns and health problems such as gastrointestinal disorders, constipation, compromised immune function and risk of food allergies and intolerances.

- Participant 001_01: … my dad is very much meat and two veg and he always has been his whole life and so was his dad before him so … and he has all sorts of bowel problems now to be honest and I think I was always very conscious of that …
- Participant 005_03: … cause if they don’t get all their vitamins they’re not going to have a very good immune system and things aren’t going to work properly …

The risk of developing food allergies and intolerances motivated one participant to form her feeding practices.

- Participant 002_03: And I also think that just with the food intolerances that people have now … my personal belief is that I would try like nuts and things and dairy and all this kind of things …

### 3.4. Challenges to Shape a Healthy Relationship with Food

Even though the time of weaning was associated with great maternal control over children’s food intake, it was also agreed that there were circumstances where this control was compromised. Some of them were directly linked to the mum, others to the children themselves, while some depended on contextual factors such as the immediate environment.

#### 3.4.1. Offering a Variety of Foods only if Mum Likes Them 

During discussions in relation to offering a variety of foods, mothers admitted that their own aversions was an inhibitor of providing a wide range of foods during weaning. Although some mums exposed their infant to foods they didn’t enjoy eating, the majority of them wouldn’t buy or offer a food that themselves disliked. Similarly, mothers tried to familiarise their children to foods they personally enjoyed eating and hence, the food consumption of their children was often a reflection of their own preferences.

- Participant 003_06: … I won’t buy things, you know, that I won’t eat.
- Participant 008_01: Yeah we tried him with everything because we liked, he liked homemade Indian and stuff like that, cause we love that sort of stuff and now like he’ll like, last night we had tikka masala.

#### 3.4.2. Misconceptions about the Definition of Food Variety

When asked how they secure a varied weaning diet, mothers demonstrated knowledge gaps in what food variety means, which was an additional barrier to providing a diverse diet. More specifically, mothers reported strategies which primarily involved exposure to a wide selection of fruit and vegetables. As quoted by one participant, including a range of herbs and spices during food preparation was also characterised as an element of food variety. None of the participants mentioned the importance of feeding foods from all food groups.

- Participant 002_05: I’ve tried different things, books, I look on the internet for recipes and things like that to see something that she will enjoy … like for example cauliflower and apple or potato and spinach and always try to have the broccoli …
- Participant 002_03: … even I when I make homemade meals I would try to like cumin and paprika, different spices and the fact that they say try to introduce everything.

#### 3.4.3. “They have Their Own Personality”

The idea that children develop their own personality early in life emerged recurrently from the data. Mothers believed that the acquisition of food likes and dislikes is an aspect of children’s temperament and hence it progressed through a personal trajectory independently from mum’s feeding practices.

- Participant 001_04: Like I tried loads of flavours within the first year and I personally don’t think it made a difference to him, cause he did try them and he ate them and then as time went on he just, just developed his own kind of little plain taste and likes what he likes and so …
- Facilitator: And how do you think this choice is formed?
  Participant 002_03: They have a little bit of their own personality. And their own taste, so they start to develop their self-awareness …
- Participant 007_04: Like even though they are young, they have their own personality and what they like and dislike.

Moreover, mothers were aware that children were born with a predisposition to sweet taste, an affinity that is responsible for their preference to sweet foods like fruit and confectionary.

- Participant 002_03: Even with the wee ones like fruit puree was always easier to get them to eat that and other stuff, so you’re already wired to sort of enjoy those things a little bit better I think.

#### 3.4.4. Being Flexible about the Feeding Environment

Mothers talked about the every-day difficulties that pose an obstacle to feeding their infants in a comfortable home environment. In relation to the location where feeding took place, some participants chose places that they considered appropriate to feed the infant, such as in the kitchen using a high chair. However, participants recognised that this was not always possible and had to be flexible depending on their activities.

- Participant 007_01: Kitchen table. The high chair when everyone else is being fed.
- Participant 005_03: But I’ll feed her anywhere, like the other day me and [infant’s name] were going into town and we were waiting on the bus and she was like right I need to feed her so I just fed her sitting at the bus stop one of her wee jars of food just feed her everywhere.

Diverging attitudes and practices were also apparent regarding the family presence during meals. Family meals were valued and viewed as a chance for family members to come together. Mothers talked about including the infant in parents’ meals even before weaning so that they become familiar with dinner time. On the other hand, eating with all the members of the family often proved challenging due to everyone’s daily activities. Often, infants were fed separately from the rest of the family due to the mismatch between parents’ schedule and infant’s routine, but also the strain put on multiparous mothers having to feed more than one child at the same time.

- Participant 008_04: … I would like that to try and do a bit more kind of around the table and bring him in so he knows this time he’s going to have something to eat ...
- Participant 007_03: I feed her first cause then she’s kind of quiet and content and try and finish the rest (of the children).
- Participant 001_04: Sometimes lunchtime would be on our own because [older baby’s name] has a big sleep and I would be trying the weaning then you know whenever I have a bit of peace and quiet.
- Participant 008_02: With (infant’s name) he would’ve sat in the kitchen in his high chair and I’d gave him his lunch and his dinner but me and my husband we sat in the living room and (infant’s name) would do like the swing and bounce and whatever or even possibly in bed by the time we were eating.

#### 3.4.5. Distractions Occurring during Feeding

It was acknowledged that infants’ restricted attention span during feeding could inhibit the completion of a meal. Mothers identified the objects that drew their children’s attention (photographs, phones, TV and tablets) and therefore, were kept away during feeding.

- Participant 001_01: Dining room for us … Anywhere else is too big of a distraction … And even then there’s exciting photographs to look at …
- Participant 008_01: … we actually started eating at the table and making a point of it and like putting all the phones down.

On the one hand, some mothers described the distractions under a positive light. For example, the TV had a pacifying effect on infants and made them more willing to open their mouths to the spoon. Similarly, in situations where the feeding didn’t progress (smoothly or at all), mothers were keen to use distractors to ease the infant into eating the food offered.

- Participant 005_03: … she stares at the TV, but I have her high chair like pointing at the TV and I’m kind of sitting in front of it so she’s still and I just put the spoon in her mouth and she just opens her mouth automatically …
- Participant 007_03: You have to make them laugh to open their mouth and it’s like ah!

It was agreed that patience is an important quality for a mother during the weaning period, as feeding takes a long time, and in certain cases, meals were even described as turbulent situations.

- Participant 002_02: But the thing is you have to be patient to feed a kid … Cause you can stay long time, ages and ages waiting on a small thing like this!
  Participant 002_04: It takes so long, it’s not like “quick eat your breakfast”!
- Participant 004_01: … and there’s some nights he just screams the place down and we’re all trying to eat and …
  Participant 004_05: Yeah it’s not a pleasant experience …

## 4. Discussion

This study employed a qualitative design to examine underexplored areas of mothers’ experiences during the complementary feeding period, particularly in relation to encouraging healthy dietary behaviours. Results elicited some of the most common opportunities mothers encounter when establishing positive eating habits, as well as relevant challenges. Independent of knowledge on infant feeding and nutrition, the participants were mindful that mothers needed to be equipped with techniques to instill a healthy relationship with food in their children. One of these techniques was being a role model of healthy eating habits during mealtimes. Participants also emphasized the importance and benefits of dietary diversity in infancy. Although the interpretation of what is a balanced and diverse diet was highly variable across the study sample, the intention to provide infants with varied dietary experiences was universal and frequently demonstrated. Distractions were seen as helpful with feeding, particularly with stressful mealtimes. 

Although mothers’ feeding approaches in certain areas diverged from each other, the findings reveal some common strategies that were used to shape a liking to healthier foods. It was evident that mothers understood that taste preferences were shaped to a large extent in infancy and used many strategies to develop taste preferences in their infant. In addition, mothers also believed that the acquisition of taste preferences was an expression of the child’s temperament, as well as inherent. These two ideas were not seen as conflicting, but in most cases as complementing each other. 

Mothers in this study played an active role in encouraging healthier food consumption and saw themselves as models encouraging positive eating behaviours in their infants. The need to be a good role model often motivated them to improve their own dietary choices, which has been highlighted in previous research [27]. According to Bandura’s Social Learning Theory, children observe and imitate behaviours, beliefs and attitudes of individuals in their immediate world such as parents [28]. Previous research findings confirm that parental modelling of dietary behaviours and attitudes can influence children’s food habits. Brown and Ogden saw a strong association between parents’ and their child’s snack intake for both healthy and unhealthy snacks [29]. Similarly, in a study by Draxten et al., children whose parents modelled eating fruit and vegetables were significantly more likely, than children whose parents did not, to meet daily fruit and vegetable recommendations [30]. These studies relate to children between 8–13 years of age, whereas the effect of modelling healthy eating behaviours in younger children is under-researched. Future research should investigate whether parental role modelling is also likely to encourage healthy eating patterns in infancy. 

Offering repeated opportunities for infants to try a certain food was regarded as an effective technique to familiarise them with this food and nurture their liking to it. Maternal confidence in this strategy aligns with previous research findings [15,31,32]. Intervention studies with infants have demonstrated that frequent exposure to a certain vegetable leads to a greater acceptance and an increased intake of that vegetable. The official recommendations in the UK and Europe have taken on board these findings and encourage repeated exposure to foods, particularly vegetables, for the development of balanced food preferences in infancy [33,34]. 

The importance of dietary diversity in the first year of life was acknowledged in this study and mothers were mindful that feeding their infants a varied diet can prevent them from becoming fussy with food, and can contribute to their good health. This finding is in line with previous qualitative evidence, where participants also explained that providing a wide range of foods and flavours during weaning was important and would help their infants adopt a healthy approach to food and avoid picky eating behaviours [35,36,37,38]. Indeed, infants fed on a variety of foods of different flavour and texture were subsequently shown to be more receptive to foods to which they had no previous exposure [32,39]. A study by Mennella et al. showed that infants who ate a variety of fruits increased their intake of a specific fruit even though they had no direct experience with this specific fruit during the exposure period in the study [39]. Furthermore, other research has shown that daily exposure to fruits enhanced the infants’ initial acceptance of a starchy vegetable (carrots) and experience with a variety of vegetables facilitated the acceptance of a novel food (chicken) [32]. 

Even though the value of feeding a variety of foods was well recognised, the maternal practices to achieve food variety revealed a limited understanding of its meaning. Dietary diversity was translated as providing a plethora of fruit and vegetables and did not address consuming foods from all food groups. The misconception that offering solely an abundance of fruit and vegetables ensures that the infant has a varied diet was not unexpected, since the recommendations on fruit and vegetables consumption in the UK are currently not met [40] and many health campaigns have targeted this message. Previous qualitative research with participants from the UK, Denmark, Germany and Sweden has also highlighted that mothers focus their efforts in feeding more fruit and vegetables during complementary feeding [27,36,41]. On the other hand, sources of high quality protein (meat, fish, pulses, eggs) were not reported by participants as elements of food variety. The omission of animal foods has been previously highlighted by the latest data of DNSIYC on food intake, where 34% of the parents reported avoiding giving all meat/poultry/fish/seafood to their 7–9 month-old children [22]. This study also revealed that maternal awareness of food allergies and intolerances plays a role in determining the diversity of the meals offered during weaning. In cases of high maternal concern about food allergies, ensuring early exposure to foods that are linked to allergic symptoms as a prophylactic measure was one of the priorities of parents’ weaning strategies. WHO also warns that complementary foods should be varied and include adequate quantities of meat, poultry, fish or eggs, as well as vitamin A-rich fruits and vegetables every day [42]. Poor parental understanding of infants’ nutritional requirements in protein and iron-rich foods can compromise dietary quality and dietary adequacy in essential amino-acids and iron (particularly in breast-fed children) with a negative impact on their nutritional status [43]. 

The period of weaning was viewed by mothers as a window of opportunity to expose children to a variety of tastes and textures. For some women, it was the only time in the children’s lifespan where the mother is in complete control over their dietary intake and therefore, weaning was associated with an urgency to introduce as many flavours as possible in this short timeframe. Similar beliefs have been demonstrated before about the importance of exposing an infant to as many foods as possible [27]. In this study, the weaning infant was also seen to be willing to try different foods. A recent review by Taylor confirms this belief and explains that unwillingness to taste new foods (neophobia) is absent in infancy, as children are experiencing food tastes and textures for the first time with no prior expectation of what they taste like [44].

The environment within which feeding takes place varied greatly. As discussed in a previous study by Brown & Lee [35], participants created occasions whereby family members shared a meal and the infant joined in. On the other hand, for the majority of the participants feeding was an additional task to an already busy schedule; this was particularly evident for women who had more than one child. For them, there was no choice but to feed their infants away from the other family members or on-the-go as part of their every-day activities. Incorporating infant feeding around daily maternal activities has been described before by Horodynski et al. [45].

Mothers in this study illustrated the clash between the ideal and reality in establishing a comfortable environment. Even though WHO encourages mums to “talk to children during feeding, with eye to eye contact”, participants here described drawing children’s attention with objects like the TV to assist with the feeding process. Employing distractions to feed was quoted as a practical way for mothers to foster positive mealtime experiences and avoid conflict over food. TV viewing during meals has been implicated in intervention studies for lessening the response to satiety cues and hence, leading to overeating [46,47]. These studies were conducted with pre-school and school-aged children, but there is no similar evidence demonstrated for children in infancy. Therefore, mealtime viewing, seen as a transient behaviour used only in infancy, should not be a cause for concern; however, it could become an issue if it forms a pathway to establishing a habit that persist later in life. Currently, the prevalence of infant feeding in front of the TV is unknown due to the scarcity of relevant data from national surveys. Feeding and TV viewing has been however described as a common maternal practice in previous qualitative literature [45]. 

### Strengths and Weaknesses

This study offers a novel perspective in relation to the underlying factors that help and impede a mother’s ability to establish healthy eating habits. The data were collected until information saturation was achieved and the broad recruitment approach enabled capturing the views of a varied sample from a range of locations and socioeconomic profiles. Nonetheless, the study findings are prone to selection bias, since the recruitment method captured the views of a predominately urban population and mothers residing in a rural setting were underrepresented. Moreover, similarly to previous studies on maternal perceptions on children’s eating behaviours, recall, perception and social desirability bias might have been introduced [48]. Additionally, during analysis a slight difference was observed in the views of mums with more than one children and first-time mums in including children in meals with the other members of the family. Further investigation with separate samples could illustrate divergent experiences between first-time and non-first-time mothers. The present study also recruited mothers who had not yet introduced non-milk foods in their infant’s diet, alongside the mothers who did. These mothers talked about their intentions in relation to weaning practices, since they hadn’t had a chance to establish any feeding practices at the time of this study, which might differ from their actual experiences later. Finally, even though the call for participation was open for both mothers and fathers, only mothers could be approached in the locations chosen during recruitment. Alternative strategies and locations need to be considered by researchers who wish to recruit fathers, which might offer a more holistic picture of the parental views and attitudes during complementary feeding [49].

## 5. Conclusions

This study presents qualitative findings on maternal approaches to nutrition during the complementary feeding period by focusing on favourable and not-so-favourable factors and situations which encourage positive eating behaviours. Mothers encouraged their children to develop a preference for healthier foods through modelling and repeated exposure to these foods. While a high perceived value for food variety was demonstrated, the definition of food variety and the methods to provide a varied and balanced weaning diet were poorly understood. Albeit deviant from infant feeding recommendations, using occasional distractions, such as the TV, were practical aids used to ease feeding without stress.

Weaning is a critical timeframe for an infant to establish healthy eating behaviours. Health care professionals in contact with new mothers, particularly health visitors, need to be aware of the common misconceptions around complementary feeding and be equipped with the appropriate knowledge and the resources to correct these misconceptions. The present study findings can be used to inform future education interventions in areas of infant nutrition that caregivers find most challenging. By addressing these areas and by approaching caregivers from a range of educational and financial status there is a great potential for current education initiatives to improve maternal feeding practices and ultimately complementary feeding.

## Figures and Tables

**Table 1:** Outline of the Focus Group Topic Guide.

Purpose/Topic	Questions/Sub Questions
Ice breaker	We can start with an introduction. Could you say your name and how old your infant is?
Key question on forming taste preferences—strategies and timing	How do you think that our likes and dislikes for foods are acquired? Can you think of an example drawn from yourselves or your children?
	Do you think that the type of foods that infants have and when they have them affects in any way their likes and dislikes and how?
	Yes—What do you do in order to shape your infant’s likes? Can you give me a few examples from your everyday experiences with your infants now or previous children?
Key question on food acceptance, exposure to novel foods	Have you ever come across cases where your infant didn’t like the foods you gave them?
	Yes—How did they express it? What kind of food(s) was/were they? Have you given your infant same foods again?
	*Show Figure*
	The children in the pictures have just been fed. How do you interpret the children’ facial expressions?
	Have you seen these expressions on your infant? Which ones and how do they affect your feeding plan?
Key question on food variety, feeding environment	How important do you think is for an infant to eat different types of foods?
	How can parents introduce food variety into their children’s diet? Add one at a time? Give a new food on its own or combined with familiar foods?
	Can you tell me a bit about the environment that you feed your infant in? Where does the feeding normally take place? Who else is there?
Ending question	Is there anything else you would like to add about weaning, especially to do with all the topics we discussed previously?

**Table 2:** Mothers’ and infants’ characteristics, *n* = 37.

Characteristics in Mean and SD	Mean ± SD	Range
Infant’s age at the time of focus group (months)	7.7 ± 3	3–16
Mother’s age at the time of focus group (year)	30.3 ± 6	19–39
Characteristics in Frequencies	*n*	%
First-time mother		
Yes/No	16/21	43.2/56.8
Mother’s ethnicity		
White	36	97.3
Black	1	2.7
Mother’s country of birth		
Northern Ireland	34	91.9
Other UK	1	2.7
Other	2	5.4
Mother’s education		
Primary school or equivalent	1	2.7
1–4 GCSEs/NVQ level 1/Foundation GNVQ/foreign equivalent	3	8.1
5+O levels/NVQ level 2/equivalent	5	13.5
Apprenticeship/2 or more A levels/NVQ level 3	9	24.3
Degree/Post-grad degree/NVQ level 4–5	19	51.4
Mother’s marital status		
Single (never married)	10	27.0
Cohabiting (living with partner)	5	13.5
Married	19	51.4
Separated	2	5.4
Divorced	1	2.7
Infant’s gender		
Girl/Boy	26/11	70.3/29.7
Breastfed at all		
Yes/No	21/16	56.8/43.2
Already on solids at the time of focus group		
Yes/No	30/7	81.1/18.9
Age of introduction of solid foods ^1^		
Up to 4mo	9	30
4–6mo	14	46.7
6 months and later	7	23.3
First solid foods given		
Baby rice and cereal products	12	32.4
Fruit	2	5.4
Vegetable	2	5.4
Fruit and vegetable	8	21.6
Mix of cereal and fruit	3	8.1
Yoghurt	2	5.4
Other	1	2.7

^1^ Frequencies expressed as proportions to women who had already weaned (as opposed to the overall sample). Abbreviations: SD: Standard Deviation; GCSE: General Certificate of Secondary Education; (G)NVQ: (General) National Vocational Qualifications.

**Table 3:** Summary of the opportunities and challenges to establish a healthy relationship with food, as emerged from the interviews (*n* = 37).

Opportunities to Shape a Healthy Relationship with Food	Challenges to Shape a Healthy Relationship with Food
1 Acting as a role model for healthy foods	1 Offering a variety of foods only if mum likes them
2 Using covert approaches to feed	2 Misconceptions about the definition of food variety
3 Giving multiple opportunities to try a food	3 “They have their own personality”
4 “It starts in the womb”	4 Being flexible about the feeding environment
5 Facial expressions not being indicative of food rejections	5 Distractions occurring during feeding
6 Food variety “so you don’t have a fussy eater”	
7 Without food variety “things aren’t going to work properly”	
